# Supplementary material for: TRIB3 supports breast cancer stemness by suppressing FOXO1 degradation and enhancing SOX2 transcription
Source: Nat Commun. 2019 Dec 16;10:5720. doi: 10.1038/s41467-019-13700-6 (PMC6915745; doi:10.1038/s41467-019-13700-6)
Supplement: Supplementary file 1 — Supplementary Info File [file 41467_2019_13700_MOESM1_ESM.pdf]

Supplementary Information

TRIB3 Supports Breast Cancer Stemness by Suppressing FOXO1 Degradation and Enhancing SOX2 Transcription

by Jin-mei Yu et al

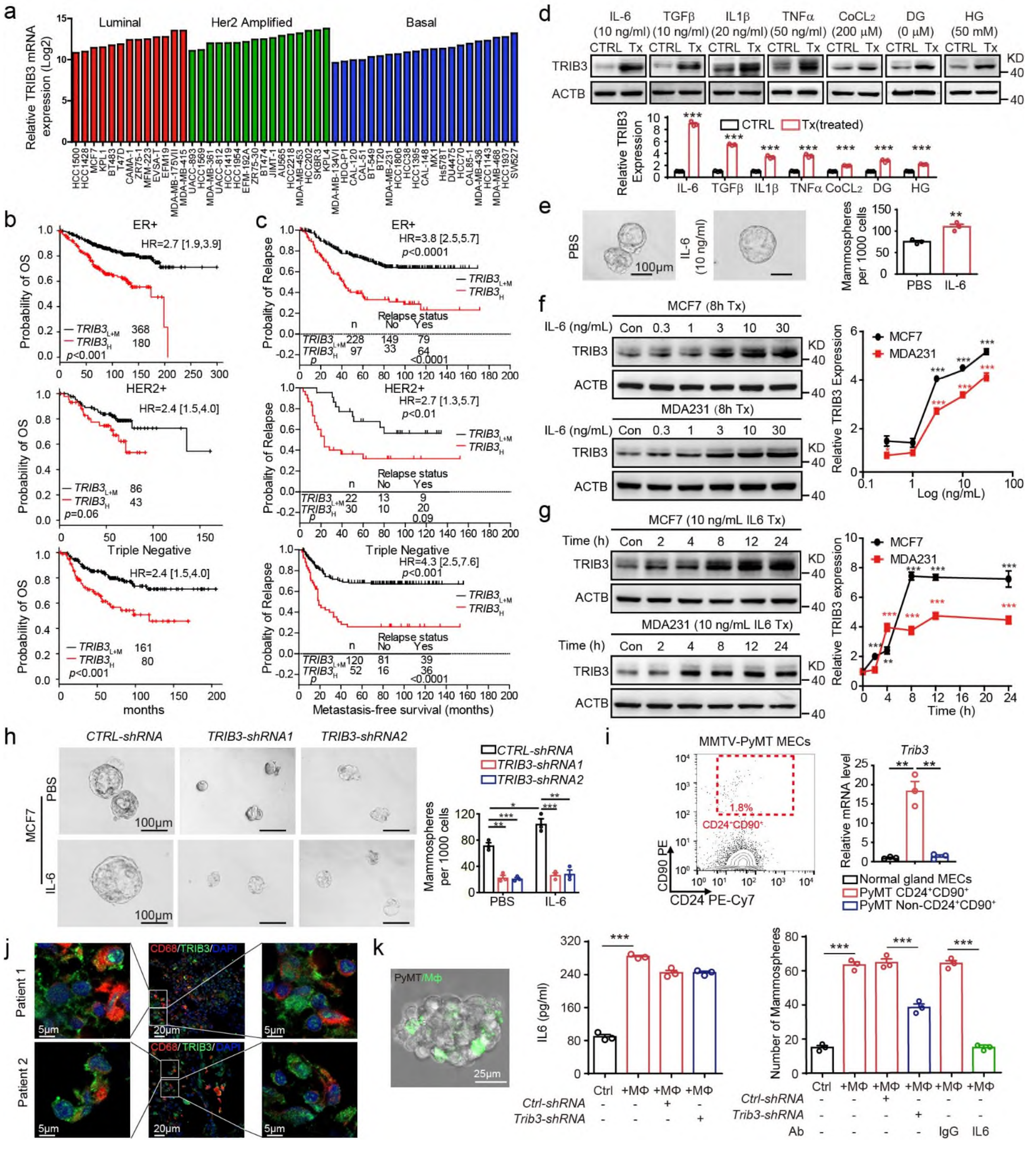

Supplementary Figure 1 High TRIB3 Expression in Breast Cancer Is Associated with Shorter Metastasis-free Survival. Related to Fig. 1. (**a**) Log2 expression values for *TRIB3* were extracted

from published gene expression data on breast cancer cells available in the GEO database (GSE12790). Relative amounts of TRIB3 transcripts were measured using the 205805_s_at probe. (**b**) (**c**) Graph derived from published data available in the PubMed GEO database (GSE2603, GSE5327, GSE2034, and GSE12276). Kaplan–Meier curves depict the prognostic impact of *TRIB3* expression on overall metastasis-free survival, lung metastasis-free survival, bone metastasis-free survival and brain metastasis-free survival. For each analysis, 582 cases were segregated into the median with the group designated $TRIB3_H$, representing half of the patients who had tumors with the highest levels of *TRIB3* mRNA, and the group designated $TRIB3_L$, representing half of patients who had cancers with the lowest levels of *TRIB3* mRNA. Metastasis-free survival was determined by Kaplan–Meier analyses, and significant differences were determined by log-rank tests. The number of patients in each category, the total metastatic events, and the corresponding *P* values (χ^2 test) are shown in the embedded tables. (**d**) The expression of TRIB3 in MCF7 cells treated with IL-6, TGFβ, IL-1β or TNFα for 8 hr, $CoCL_2$ for 24 hr, glucose deprivation (DG) for 12 hr, and high glucose (HG) for 24 hr was detected by Western blotting (WB). (**e**) Tumor sphere formation of MCF7 cells after one week of PBS or IL-6 treatment (as indicated on the left) in serum-free spheroid suspension culture conditions. (**f, g**) The expression of TRIB3 in MCF7 cells (top) or MDA-MB-231 cells (bottom) treated with various concentrations of IL6 for 8 hr (f) or 10 ng/ml IL-6 for different amounts of time (g) was detected by WB. (**h**) Silencing TRIB3 decreased the tumor sphere formation ability of MCF7 cells with or without IL-6 stimulation. (**i**) Flow cytometry of Lin^- MECs from the mammary glands of 6-week-old MMTV-PyMT female mice. The relative *Trib3* mRNA expression is indicated in the histograms at the right. (**j**) Immunofluorescence indicates high TRIB3 (green) expression adjacent to $CD68^+$ (red) TAMs from breast cancer patients. (**k**) Co-culture mammosphere assay of $CD45^-CD31^-TER119^-(Lin^-)$ mammary epithelial cells (MECs) with $CD45^+F4/80^+CD11b^+Gr1^-$ macrophages (green) from 10-week-old MMTV-PyMT female mice (left). Quantification of IL6 production in control MECs, *Ctrl-shRNA* MECs, and *Trib3-shRNA* MECs with or without macrophages co-culture (middle). Histogram graphs showing numbers of mammospheres formed by control MECs with or without macrophages, *Ctrl-shRNA* MECs, *Trib3-shRNA* MECs, and control MECs treated with the indicated antibody with macrophages, respectively (right). n=3 samples. Data are shown as the mean ± SEM; *P*>0.05 was considered not significant (N.S.); **P*<0.05, ***P*<0.01 and ****P*<0.001, compared with the con group in panels d-f. Source data are provided as a Source Data file.

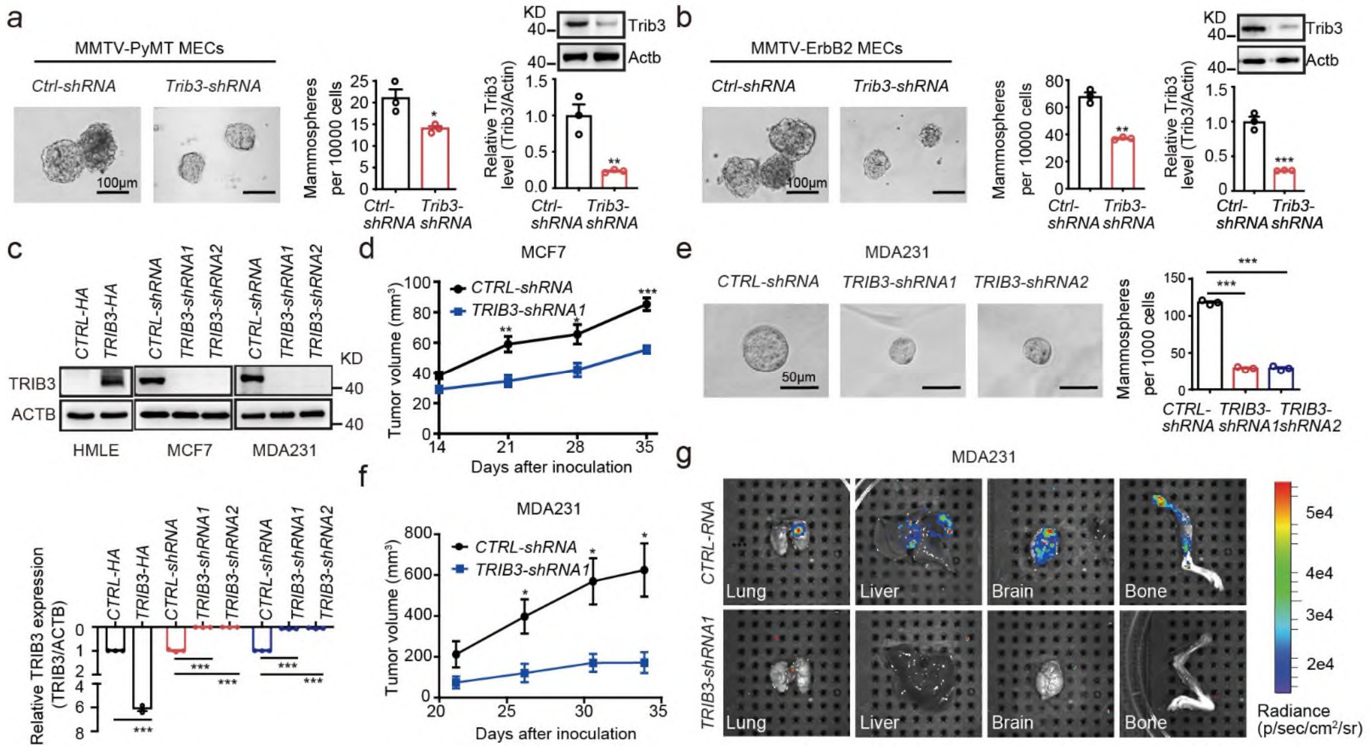

Supplementary Figure 2 Silencing TRIB3 Inhibits Spheroid Formation, Tumor Proliferation and Metastasis. Related to Fig. 2. (**a, b**) Tumor spheroid formation of CD45$^-$CD31$^-$TER119$^-$ (Lin$^-$) mammary epithelial cells (MECs) from the mammary glands of 6-week-old MMTV-PyMT female mice (a) or 6-month-old MMTV-Erbb2 female mice (b). Mammosphere quantification and relative Trib3 protein expression are indicated in the right panels. (**c**) Immunoblots of protein lysates of cells, as indicated at the bottom, transfected with a control vector or a *TRIB3*-expressing vector for HMLE cells or with *CTRL-shRNA* or *TRIB3*-shRNA for MCF7 and MDA-MB-231 cells. (**d**) NOD-SCID mice were administered 6x10^5 *CTRL-shRNA*-transfected (black) or *TRIB3-shRNA*-transfected (blue) MCF7 cells via subcutaneous (s.c.) injection into their mammary fat pads (n=5 per group). The primary tumor in the injected mammary fat pad was measured at various times after engraftment, as indicated at the bottom. (**e**) Tumor spheroid formation of MDA-MB-231 cells transfected with either *CTRL-shRNA* or *TRIB3-shRNA*. Data are shown as the number of mammospheres per 1000 cells (right panel). (**f**) NOD-SCID mice were administered 2.5x10^4 *CTRL-shRNA*-transfected (black) or *TRIB3-shRNA*-transfected (blue) MDA-MB-231 cells via s.c. injection into their mammary fat pads (n=5 per group). The primary tumor in the injected mammary fat pad was measured at various times after engraftment, as indicated at the bottom. (**g**) Representative bioluminescence imaging of lung, liver, brain and bone from NOD-SCID mice engrafted with 1x10^6 luciferase-labeled MDA-MB-231 cells transduced with *CTRL-shRNA* (top row) or *TRIB3-shRNA1* (bottom row). The intensity of the fluorescence signal, related to the cell number,

is indicated by the scale on the right. Data are shown as the mean ± SEM; *P*>0.05 was considered not significant (N.S.); **P*<0.05, ***P*<0.01, and ****P*<0.001, compared with the vector or *CTRL-shRNA* group. Source data are provided as a Source Data file..

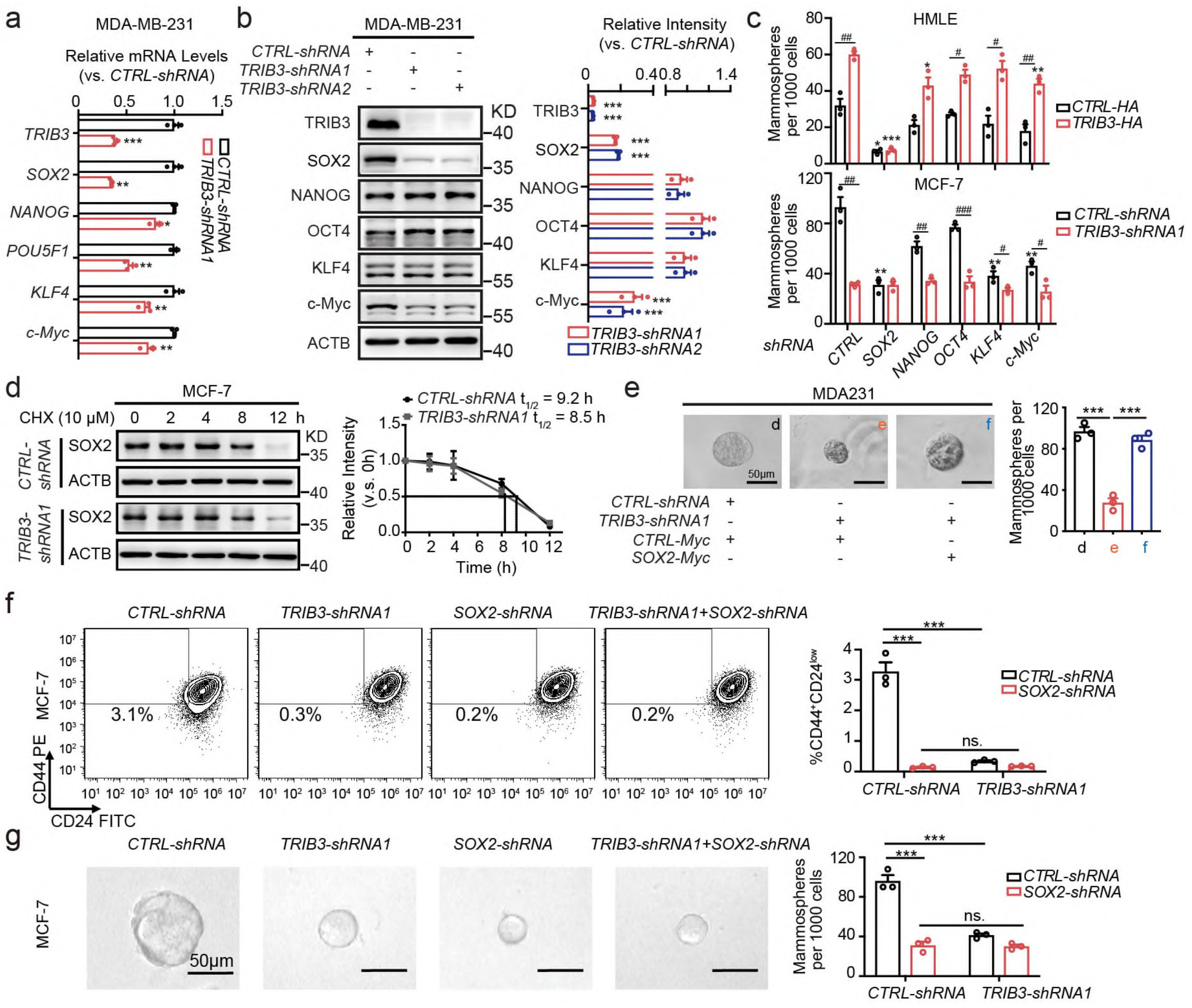

Supplementary Figure 3 TRIB3 Enhances SOX2 Expression in Breast Cancer Cells. Related to Fig. 3. (**a**) Histogram showing the expression of *TRIB3* (far left) and stem cell-related genes in MDA-MB-231 cells transfected with *TRIB3-shRNA1* vs. *CTRL-shRNA*. (**b**) Immunoblots and statistical analysis of protein lysates of MDA-MB-231 cells, as indicated on the left, transfected with *CTRL-shRNA* or *TRIB3*-shRNA. (**c**) Tumor spheroid formation of HMLE-*CTRL*, HMLE-*TRIB3*, MCF7-*CTRL-shRNA* or MCF7-*TRIB3-shRNA* cells transfected with *CTRL-shRNA*, *SOX2-shRNA, NANOG-shRNA, OCT4-shRNA, KLF4-shRNA* or *c-Myc-shRNA*. (**d**) Control or TRIB3-silenced MCF7 cells were incubated with CHX (cycloheximide) for the indicated amounts of time. Cell lysates were isolated for immunoblotting. (**e**) Tumor spheroid formation of MDA-MB-231 cells transfected with *CTRL-shRNA* or *TRIB3-shRNA* and either a control vector or a SOX2-expressing vector. (**f**) Flow cytometric assays detecting the

percentage of CD44^{Br}CD24^{Di} in MCF7 cells transfected with *CTRL-shRNA*, *TRIB3-shRNA*, *SOX2-shRNA* or *TRIB3-shRNA* plus *SOX2-shRNA*. (**g**) Tumor spheroid formation of MCF7 cells transfected with *CTRL-shRNA*, *TRIB3-shRNA*, *SOX2-shRNA* or *TRIB3-shRNA* plus *SOX2-shRNA*. Data are shown as the mean ± SEM of three independent assays. *P*>0.05 was considered not significant (N.S.); **P*<0.05, ***P*<0.01, and ****P*<0.001 compared with the vector or *CTRL-shRNA* group. Source data are provided as a Source Data file..

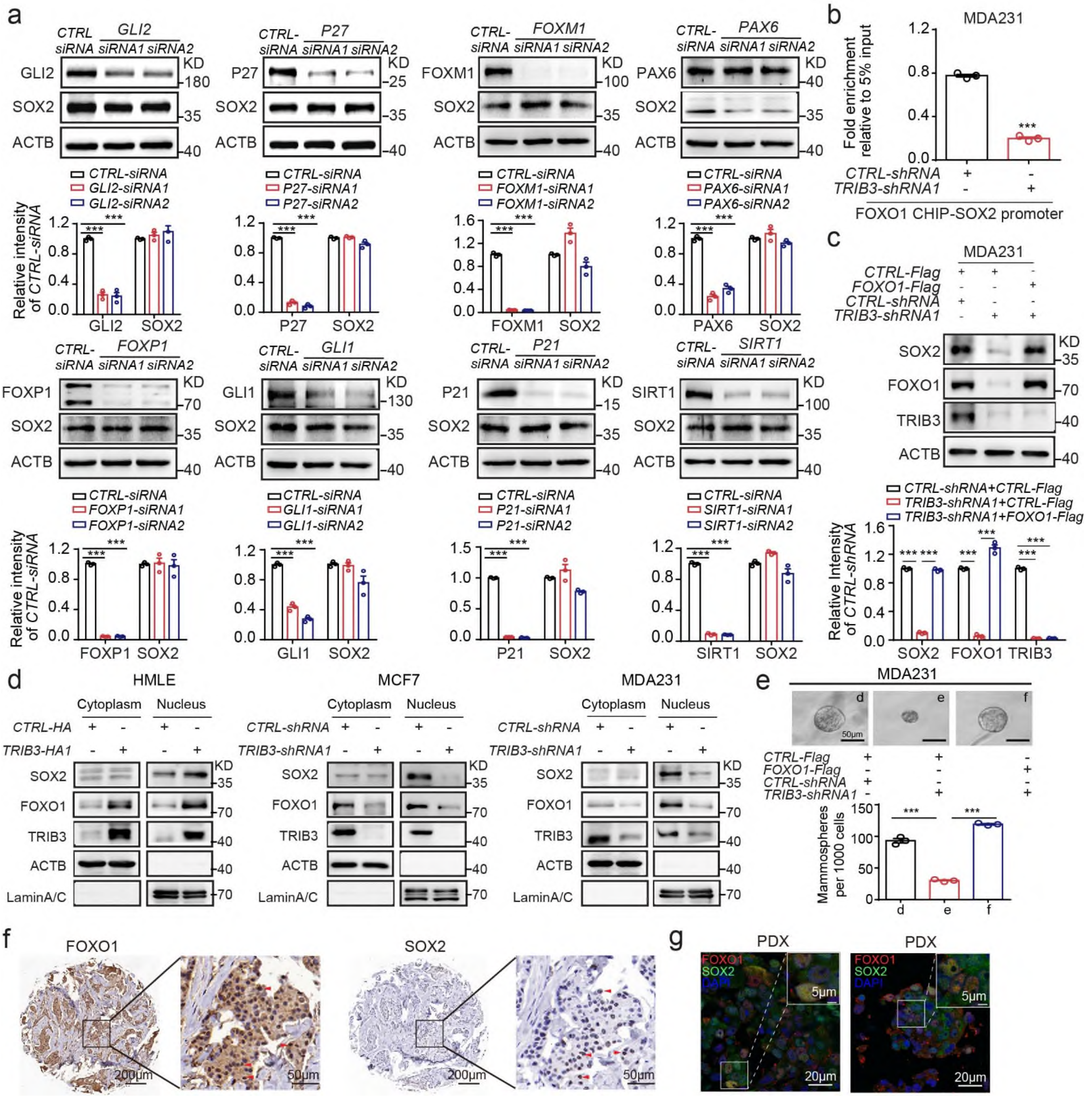

Supplementary Figure 4 Transcription Factor Screening for the TRIB3-SOX2 Axis in Breast Cancer Cells. Related to Fig. 4. (a) Sample immunoblots and quantitative analyses of SOX2 expression after silencing the indicated SOX2 transcription factors, including GLI2, P27, FOXM1, PAX6, FOXP1, GLI1, P21 and SIRT1, in MCF7 cells (n = 3). β-actin was used as a loading control for immunoblotting. (**b**) FOXO1 ChIP-SOX2 promoter activity in MDA-MB-231 cells transfected with either

CTRL-shRNA or *TRIB3-shRNA*. (**c**) Immunoblots and statistical analysis of protein lysates from MDA-MB-231 cells transfected with *CTRL-shRNA* or *TRIB3-shRNA* and either a control vector or a FOXO1-expressing vector. (**d**) Immunoblots of cytoplasmic and nuclear protein lysates of HMLE cells transfected with a control vector or a TRIB3-expressing vector or MCF7 cells and MDA-MB-231 cells transfected with *CTRL-shRNA* or *TRIB3-shRNA*. (**e**) Tumor spheroid formation of MDA-MB-231 cells transfected with *CTRL-shRNA* or *TRIB3-shRNA* and either a control vector or a FOXO1-expressing vector. (**f**) Formalin-fixed, paraffin-embedded tissue microarray sections of breast cancer tissues were stained with an anti-SOX2 antibody and an anti-FOXO1 antibody. $SOX2^{+}FOXO1^{+}$ double positive breast cancer cells are indicated with red arrows. (**g**) Frozen breast cancer patient-derived xenograft (PDX) sections were stained with anti-SOX2 (green) and anti-FOXO1 (red) antibodies. $SOX2^{+}FOXO1^{+}$ double-positive breast cancer cells are shown with yellow color. Data are shown as the mean ± SEM; P>0.05 was considered not significant (N.S.); *P<0.05, **P<0.01, and ***P<0.001 compared with the vector or *CTRL-shRNA* group. Source data are provided as a Source Data file.

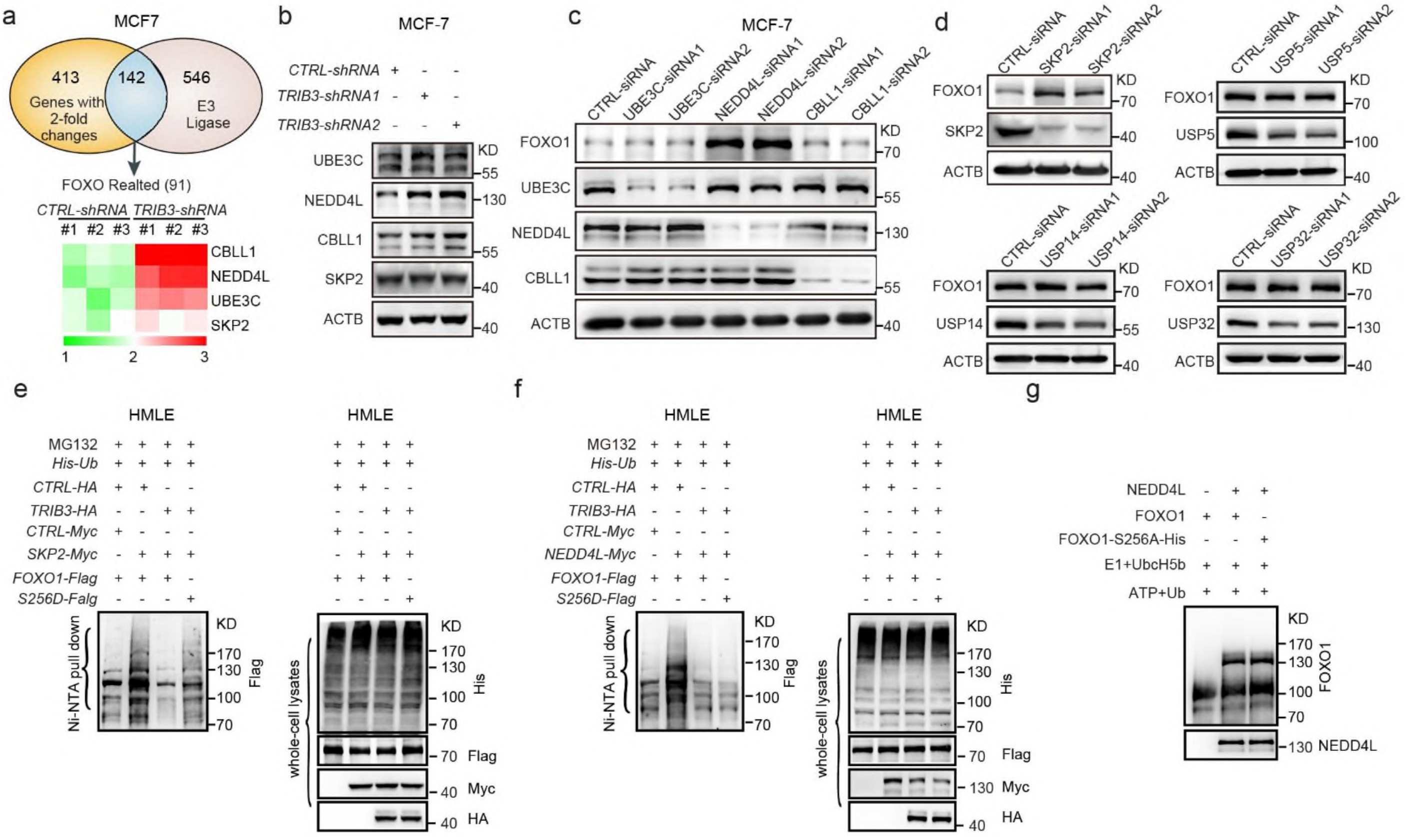

Supplementary Figure 5 TRIB3 Reduces NEDD4L Expression and Inhibits SKP2 and NEDD4L Mediated FOXO1 Degradation. Related to Fig. 5. (a) Schematic diagrams of FOXO1-related E3 ligases after TRIB3 silencing. Among the 91 FOXO-related E3 ligase genes, *CBLL1*, *NEDD4L*, *UBE3C* and *SKP2* were the most abundantly altered by TRIB3 silencing. (**b**) Immunoblots and statistical analysis of protein lysates of MCF7 cells, as indicated on the left, transfected with *CTRL-shRNA* or *TRIB3-shRNA*. (**c-d**) Sample immunoblots of FOXO1 expression in MCF7 cells after silencing the indicated E3 ubiquitin ligases, including UBE3C (c), NEDD4L (c), CBLL1 (c) and SKP2 (d), or deubiquitinating enzymes, including USP5 (d), USP14(d) and USP32 (d). (**e-f**) The effect of TRIB3 on FOXO1 ubiquitylation by SKP2 (e) or NEDD4L (f). HMLE cells were transfected with the indicated plasmid. His-ubiquitin-conjugated proteins were pulled down from cell lysates. Total protein lysates and Ni-NTA-agarose eluates were Western blotted and stained for FLAG, His, Myc, and HA. (**g**) *In vitro* ubiquitination of FOXO1 by NEDD4L. Purified recombinant FOXO1 protein or FOXO1-S256A protein was incubated with purified E1, E2 UbcH5b and E3 ligase NEDD4L as indicated. The reaction was subjected to anti-FOXO1 and anti-NEDD4L immunoblotting. The source data are provided in the Source Data file.

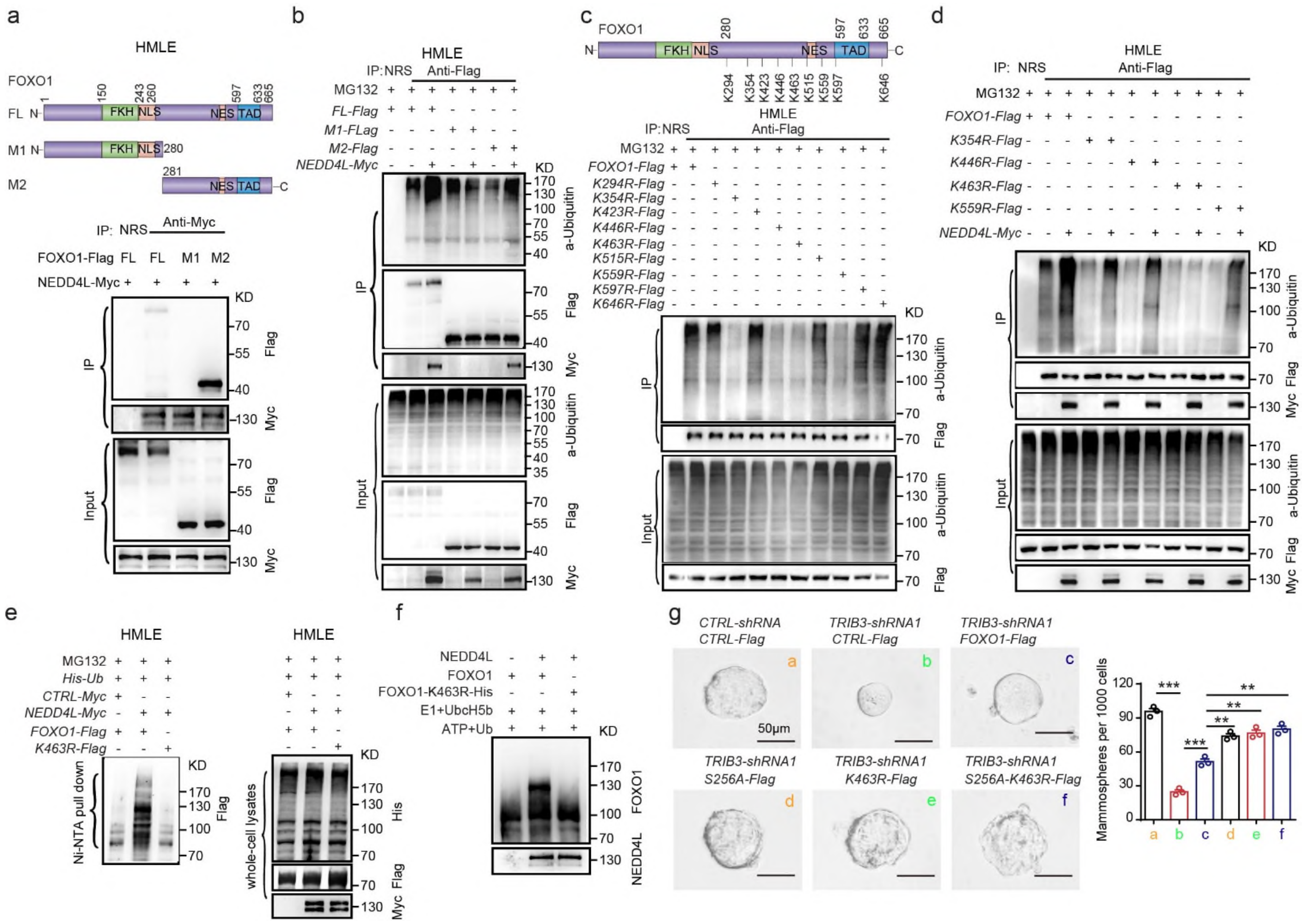

Supplementary Figure 6 TRIB3 reduces NEDD4L expression and inhibits SKP2 and NEDD4L-mediated FOXO1 degradation. Related to Fig. 5. (**a-b**) Mapping FOXO1 regions binding to NEDD4L. Schematic diagram of full-length FOXO1 and deletion mutants (a, top). HMLE cells were co-transfected with the indicated FOXO1-Flag and NEDD4L-Myc constructs. Protein interactions (a, bottom) and FOXO1-Flag ubiquitylation (b) were detected in cell extracts immunoprecipitated (IP) with an anti-Myc or anti-Flag Ab. (**c**) Mapping the lysine sites of the FOXO1 M2 domain in HMLE cells. Top: schematic of FOXO1 deletion mutant lysine sites. Bottom: mutants of FOXO1 ubiquitylation in HMLE cells. Cell extracts were IP with an anti-Flag Ab. (**d**) Mapping the key lysine sites of FOXO1 ubiquitylation by NEDD4L in HMLE cells. Cell extracts were IP with an anti-Flag Ab. (**e**) The effect of K463 on FOXO1 ubiquitylation by NEDD4L. The ubiquitinated FOXO1-Flag was detected by immunoblotting. HMLE cells were transfected with the indicated plasmid. His-ubiquitin-conjugated proteins were pulled down from cell lysates. Total protein lysates and Ni-NTA-agarose eluates were immunoblotted and stained for FLAG, His, and Myc. (**f**) *In vitro* ubiquitination of FOXO1 by NEDD4L. Purified recombinant FOXO1

protein or FOXO1-K463R protein was incubated with purified E1, E2 UbcH5b and E3 ligase NEDD4L as indicated. The reaction was subjected to anti-FOXO1 and anti-NEDD4L immunoblotting. (**g**) Tumor spheroid formation of MCF7 cells transfected with a control vector or a TRIB3-expressing vector and either *CTRL-Flag, FOXO1-Flag* or mutated *FOXO1-Flag* (as indicated at the top). Data are shown as the mean ± SEM; *P*>0.05 was considered not significant (N.S.); **P*<0.05, ***P*<0.01, ****P*<0.001 compared with the vector or *CTRL-shRNA* group. Source data are provided as a Source Data file.

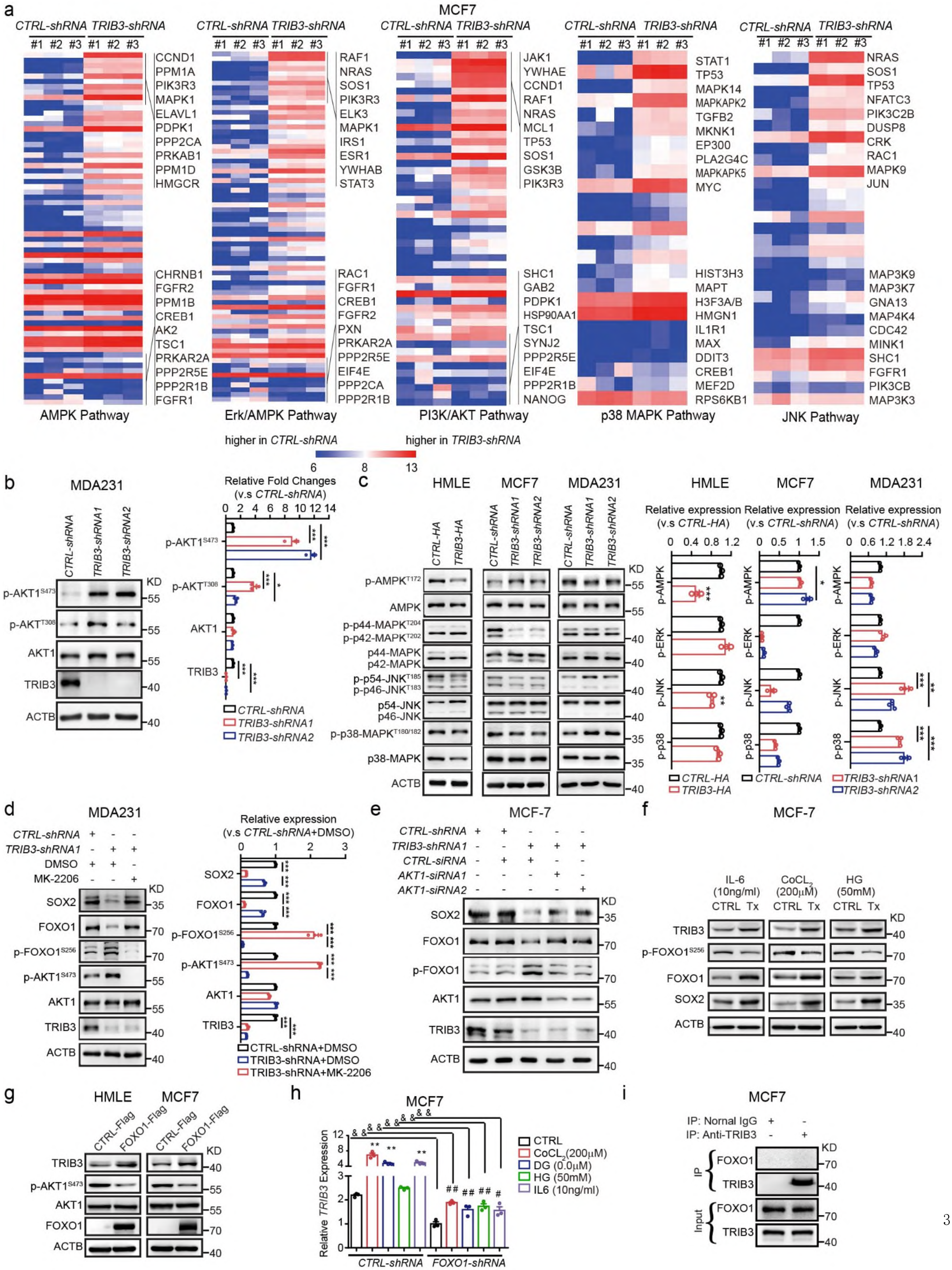

MCF7
AMPK Pathway
Erk/AMPK Pathway
PI3K/AKT Pathway
p38 MAPK Pathway
JNK Pathway
higher in CTRL-shRNA
higher in TRIB3-shRNA
MDA231
HMLE
MCF7
MCF-7

Supplementary Figure 7 AKT, but not Other Pathways, Mediated the TRIB3-FOXO1-SOX2 axis. Related to Fig. 6. (a) Each column represents a separate group of MCF7 cells transfected with *CTRL-shRNA* or *TRIB3-shRNA*, as indicated at the top. The relative expression of the genes indicated in the right margin is provided in rows using the color coding for log_2 normalized values according to the scale provided at the bottom of each heat map. (**b**) TRIB3 inhibited the activity of AKT1 in MDA-MB-231 breast cancer cells. The indicated stable expression cell extracts were prepared, and the levels of the indicated proteins were detected by immunoblotting. (**c**) The effects of TRIB3 on the activity of AMPK, ERK, p38 MAPK and JNK in breast cancer cells. (**d**) The effects of AKT1 antagonist MK2206 for TRIB3-FOXO1-SOX2 axis in MDA-MD-231 cells. MDA-MB-231 cells were treated with the AKT1 antagonist MK2206 for 24 hr. Cell extracts were prepared and the levels of the indicated proteins were detected by immunoblotting. (**e**) The effects of silencing AKT1 for TRIB3-FOXO1-SOX2 axis in MCF7 cells. MCF7 cells were transfected with the indicated siRNAs for 48 hr. Cell extracts were prepared, and the levels of the indicated proteins were detected by immunoblotting. (**f**) The expression of the TRIB3/AKT/FOXO1/SOX2 axis in MCF7 cells treated with IL-6 for 12hr, $CoCL_2$ for 24 hr, and high glucose (HG) for 24 hr was detected by Western blotting. (**g**) Overexpression FOXO1 enhanced TRIB3 expression in HMLE and MCF7 cells. (**h**) The relative *TRIB3* mRNA expression of MCF7 cells transfected with *CTRL-shRNA* or *FOXO1-shRNA* by IL-6, $CoCL_2$, glucose deprivation (DG), or HG treatment. (**i**) TRIB3 has no interaction with FOXO1 in MCF7 cells. MCF7 cell extracts were IP with an anti-TRIB3 Ab or normal IgG. Source data are provided as a Source Data file.

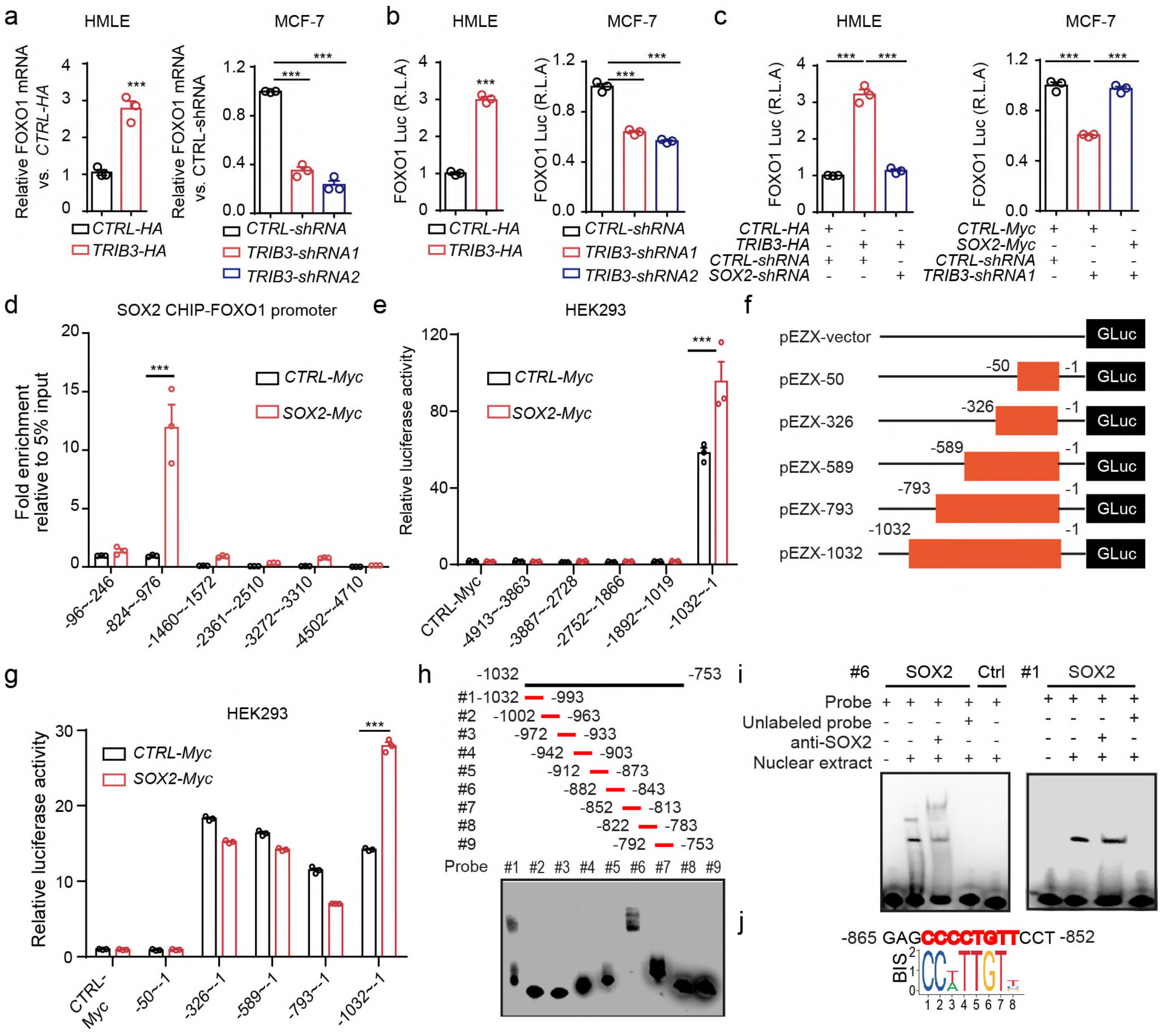

Supplementary Figure 8 SOX2 Enhances FOXO1 Transcription by Binding the FOXO1 Promoter. Related to Fig. 6. (a-b) Relative *FOXO1* gene expression (a) and FOXO1 promoter luciferase activity (b) in HMLE cells (left) transfected with a control vector or a TRIB3-expressing vector or in MCF7 cells (right) transfected with *CTRL-shRNA* or *TRIB3-shRNA* and either a control vector or a FOXO1-expressing vector. (**c**) FOXO1 transcriptional activity in HMLE cells (left) transfected with a control vector or a TRIB3-expressing vector and either *CTRL-shRNA* or SOX2-*shRNA* (as indicated at the bottom) or in MCF7 cells (right) transfected with *CTRL-shRNA* or *TRIB3-shRNA* and either a control vector or a SOX2-expressing vector. (**d**) SOX2 is recruited to the FOXO1 promoter. ChIP analysis was used to monitor the binding of SOX2 to the FOXO1 promoter in HEK293 cells expressing an empty

vector (CTRL) or a SOX2-Myc vector. Data represent at least three separate experiments and are shown as the mean ± SEM. ***P < 0.001. (**e-g**) SOX2 enhances FOXO1 transcription. Different loci of the FOXO1 promoter were inserted into the pEZX vector, and the constructs were transfected into HEK293 cells expressing an empty vector (Ctrl) or SOX2 and then subjected to a luciferase reporter assay. Data are representative of three different experiments and are shown as the mean ± SEM. **P < 0.01. (**h**) SOX2 binds to the -882~-843 segment of the FOXO1 promoter. Nuclear extracts from HEK293 cells expressing SOX2 were monitored by EMSA assay with different probes as shown in the upper panel. (**i**) SOX2 binds a specific locus on the FOXO1 promoter. The EMSA assay was used to monitor SOX2 binding to the FOXO1 promoter with a specific 15-nucleotide probe (#6 probe, as in h) with nuclear extracts from HEK293 cells expressing an empty vector (Ctrl) or SOX2. An anti-SOX2 antibody was used in the supershift assay, and unlabeled probes were used for the competitive reaction. (**j**) Prediction of SOX2 binding sites on the FOXO1 promoter from JASPAR database. Source data are provided as a Source Data file.

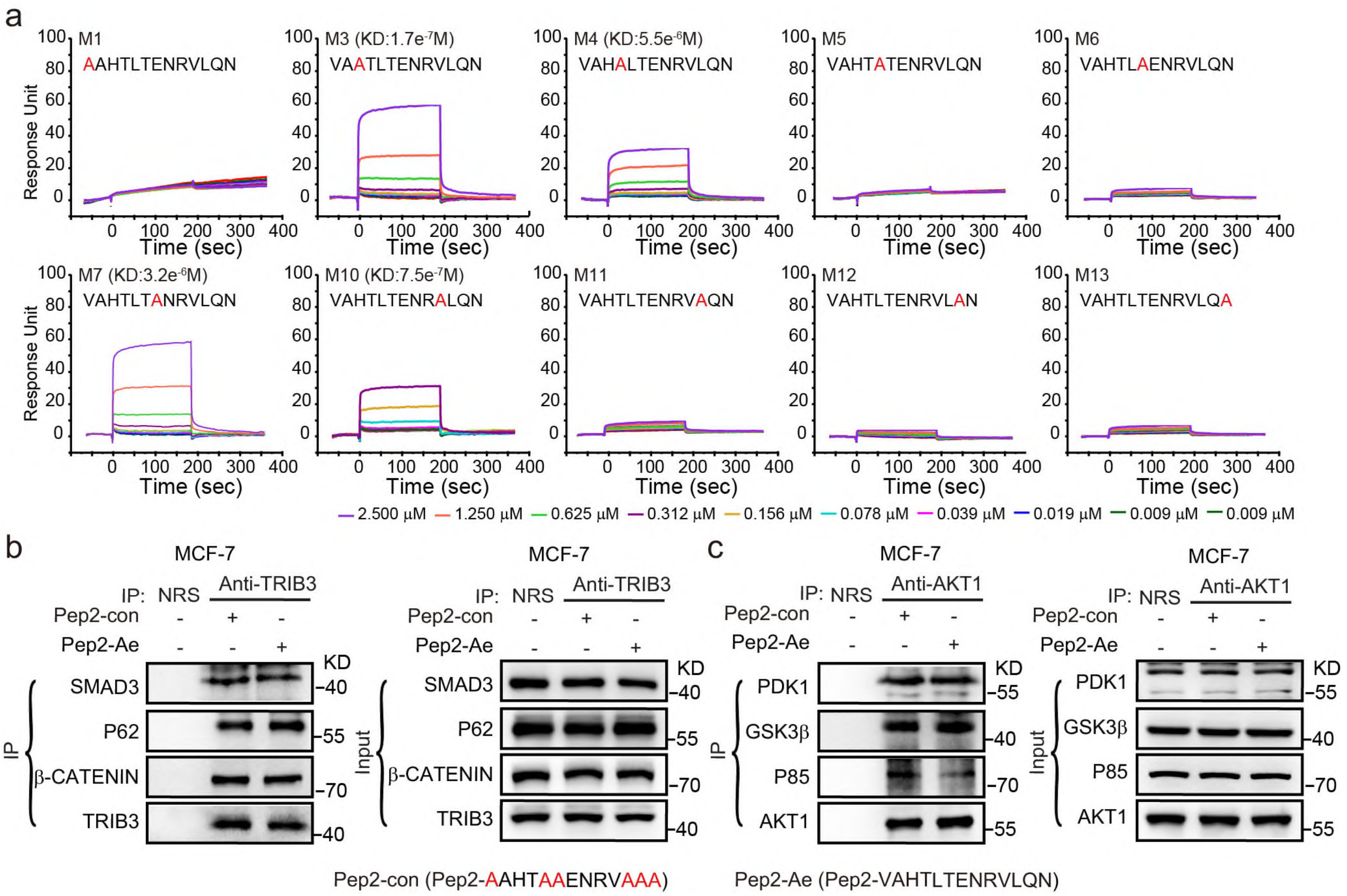

Supplementary Figure 9 Key Amino Acid Identification and Specificity of Pep2–Ae by SPR and Immunoprecipitation. Related to Fig 7. (**a**) Each amino acid of peptide Ae was substituted with alanine. Kinetic interactions of mutated peptides and TRIB3 were determined by SPR analyses. (**b**) Pep2–Ae has no effect on the TRIB3/SMAD3, TRIB3/P62, and TRIB3/β-CATENIN interactions. (**c**) Pep2-Ae has no effect on the AKT1/PDK1, AKT1/GSK3β, and AKT1/P85 interactions. Source data are provided as a Source Data file.

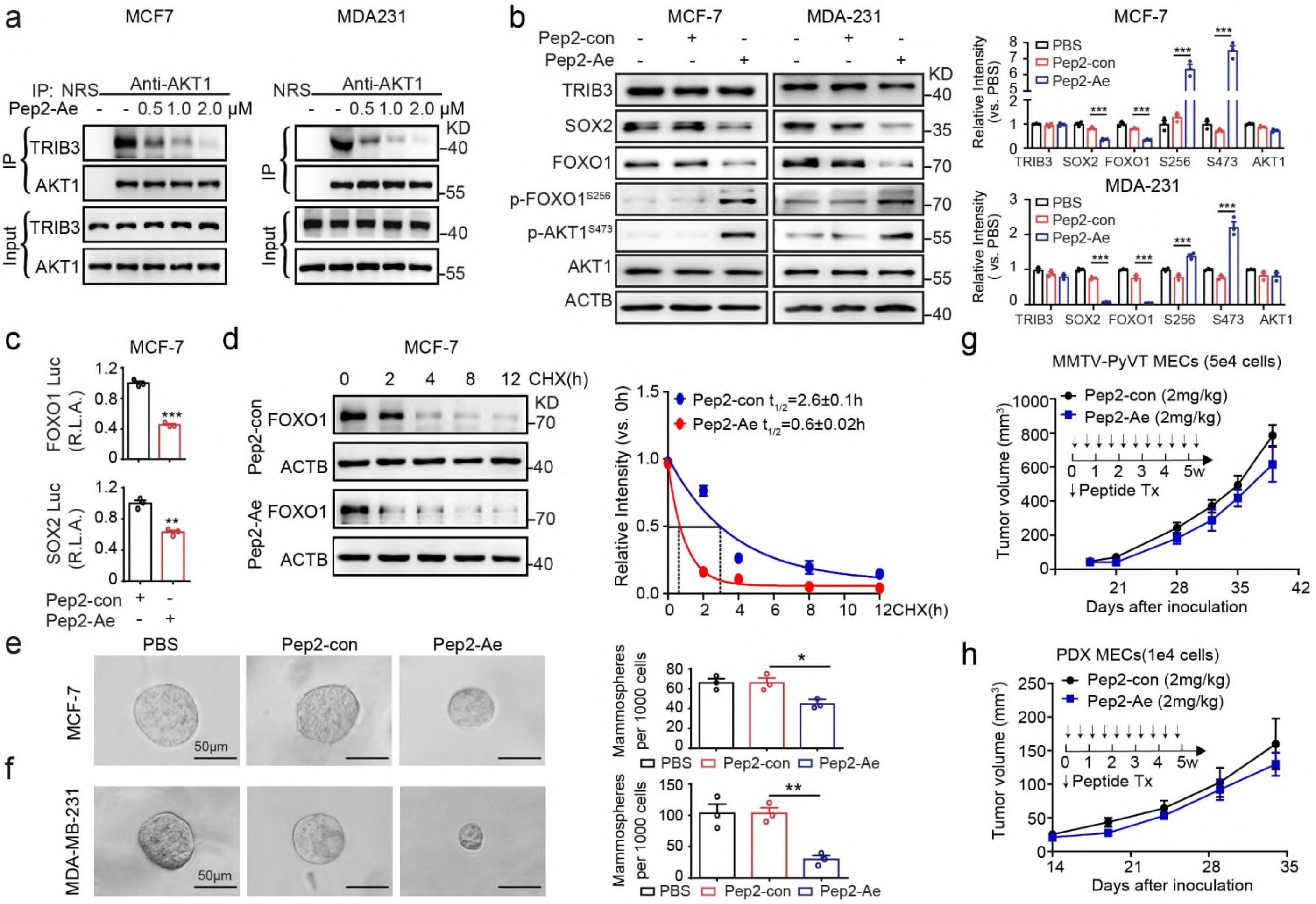

Supplementary Figure 10 Pep2–Ae Breaks the TRIB3-AKT Interaction in Breast Cancer Cells. Related to Fig 8. (**a**) Pep2–Ae treatment disturbed the TRIB3/AKT1 interaction. MCF7 and MDA-MB-231 cells were treated with the indicated peptides. Tumor cells were IP with an anti-AKT1 Ab and blotted with an anti-TRIB3 Ab. (**b**) The effect of Pep2–Ae on the TRIB3/FOXO1/SOX2 axis. MCF7 or MDA-MB-231 cells were treated with the indicated peptides (1 μM). Cell extracts were blotted with the indicated Abs. (**c**) The effect of Pep2–Ae on the promoter activity of FOXO1 and SOX2. MCF7 cells were transfected with either pEZX-PG04- FOXO1 or pEZX-PG04-SOX2. After 24 hr, MCF7 cells were treated with the indicated peptides (1 μM) for 24 hr and luciferase activity was measured. (**d**) Pep2–Ae enhanced FOXO1 degradation. MCF7 cells were treated with 20 μM CHX and the indicated peptides for different amounts of times. The indicated proteins were detected with immunoblotting. (**e-f**) Pep2–Ae inhibited mammosphere formation. MCF7 and MDA-MB-231 cells were cultured in tumor sphere medium and treated with 1 μM the indicated peptides for 5-7 days. The size and the number of mammospheres were analyzed. (**g-h**) FVB mice were given subcutaneous (s.c.) injections of MMTV-

PyVT MECs (g). NSG mice were given s.c. injections of PDX MECs (h). Mice were treated with Pep2-Ae or control peptides twice a week for 5-6 weeks. The tumor volume was calculated as 0.5 x Length x Width^2. The MECs from peptide-treated tumors were isolated, and secondary transplantation was conducted. Data are shown as the mean ± SEM; *P*>0.05 is considered not significant (N.S.); **P*<0.05, ***P*<0.01, and ****P*<0.001 compared with Pep2–con group. Source data are provided as a Source Data file.

Supplementary Table 1. Correlation of *TRIB3* status with various clinical features of breast cancer

		TRIB3$_H$	*TRIB3*$_M$	*TRIB3*$_L$	*p*-value
ER	ER+	97	127	101	0.01
	ER^{Neg}	86	65	91	
	NA	11	2	2	
PR	PR+	75	114	82	0.001
	PR^{Neg}	101	75	98	
	NA	22	5	10	
HER2	HER2+	52	34	20	<0.001
	$HER2^{Neg}$	115	134	155	
	NA	34	21	17	
Triple Negative	$ER^{Neg}PR^{Neg}$ $HER2^{Neg}$	47	31	65	<0.001
	Others	114	135	101	
	NA	33	28	28	
T-Stage	T1	52	79	69	0.001
	T2	93	72	80	
	T3	17	6	3	
	T4	5	2	2	
	NA	26	25	51	
Size (mm)		27.2	18.2	18.1	<0.001
Age		50.3	54.4	52.3	0.007

The table was derived from published data available through the PubMed GEO database (GSE2603, GSE5327, GSE2034, and GSE12276). A total of 582 cases were segregated into tertiles with the *TRIB3*$_H$ group, *TRIB3*$_M$ group or *TRIB3*$_L$ group based on *TRIB3* mRNA expression. For analysis of estrogen receptor (ER), progesterone receptor (PR), HER2, triple negative and T-Stage by TNM classification, p-values were calculated based on the Pearson Chi-Square Test. For tumor size and age of patients, p-values were calculated based on One Way ANOVA test. NA = not available.

Supplementary Table 2. Cox regression analysis of metastasis-free survival (Cox proportional hazards regression model) of patients with breast cancer

A. Univariate

Predictor	Overall Relapse			Lung Relapse			Bone Relapse		
	HR	95% CI	*P*	HR	95% CI	*P*	HR	95% CI	*P*
ER	0.8	0.6-1.1	0.1	0.3	0.2-0.5	<0.001	1.0	0.7-1.4	0.8
HER2	1.2	0.9-1.7	0.2	0.9	0.5-1.6	0.7	1.4	0.9-2.1	0.09
Triple Negative	1.2	0.9-1.7	0.1	2.8	1.7-4.5	<0.001	1.0	0.7-1.6	0.9
$TRIB3_H$	3.2	2.5-4.1	<0.001	3.3	2.1-5.1	<0.001	3.0	2.2-4.2	<0.001

B. Multivariable

Predictor	Overall Relapse			Lung Relapse			Bone Relapse		
	HR	95% CI	*P*	HR	95% CI	*P*	HR	95% CI	*P*
ER	1.2	0.7-1.9	0.41	0.4	0.2-1.9	0.06	1.2	0.6-2.3	0.6
HER2	1.2	0.8-1.8	0.35	0.8	0.3-1.8	0.5	1.3	0.8-2.1	0.3
Triple Negative	1.6	1.0-2.7	0.07	1.5	0.6-3.5	0.37	1.3	0.6-2.7	0.5
$TRIB3_H$	3.0	2.3-4.0	<0.001	2.7	1.6-4.4	<0.001	2.8	2.0-4.0	<0.001

The table was derived from published data available through the PubMed GEO database (GSE2603, GSE5327, GSE2034, and GSE12276). A total of 582 cases were segregated into tertiles with the $TRIB3_H$ (high) group, $TRIB3_M$ (intermediate) group or $TRIB3_L$ (low) group based on *TRIB3* mRNA expression. The performance of $TRIB3_H$ in predicting metastasis-free survival was analyzed by multivariate analyses with Cox proportional hazard regression models. The hazard ratio of each covariate and its 95% confidence interval are reported. *p*-values were calculated based on the normal distribution, assessing the probability of the null hypothesis (hazard ratio = 1, i.e., no prognostic significance) to be true.

Supplementary Table 3 Patient-related information. Related to Fig. 8 and Supplementary Figure 1,4 and 10

ID	Age	Sex	Tumor Site	Cancer Type	IHC staining
Patient 1	51	Female	Breast	Triple-negative	ER(-), PR(-), HR(-)
Patient 2	53	Female	Breast	Luminal B	ER(+), PR(+), HR(-)
Patient 3	57	Female	Breast	Triple-negative	ER(-), PR(-), HR(-)

Supplementary Table 4 Primer Sequences for qRT-PCR and EMSA. Related to Fig. 1-4 and Supplementary Figure 1, 3, 6 and 8.

Gene name	Primer sequences	
	Forward	Reverse
Trib3	CCATTTGGTCCTGACGGAAAGTCG	AGCCTTGAAGTCACAAGCCGTTTC
Gapdh	GGTCCCAGCTTAGGTTCATCAGGT	AATACGGCCAAATCCGTTCACACC
TRIB3	CCATTTGGTCCTGACGGAAAGTCG	AGCCTTGAAGTCACAAGCCGTTTC
SOX2	AGCTACAGCATGATGCAGGACCAG	CGTTCATGTAGGTCTGCGAGCTG
POU5F1	TTTTGGTACCCCAGGCTATGGGAG	GTTTGAATGCATGGGAGAGCCCAG
NANOG	ACTCTTCCTACCACCAGGGATGC	CTCCAGGACTGGATGTTCTGGGTC
KLF4	AAGGTCAGTCCCGGGGATTTGTAG	GAGCTCTAGGGGTGAAGAAGGTGG
C-Myc	CGGGTAGTGGAAAACCAGAGGAGG	CCAGACTCTGACCTTTTGCCAGGAG
FOXO1	TGGAGATCGACCCGGACTTCGAG	GTGGCCGAGTTGGACTGGCTAAAC
SOX2 (ChIP)	ATGCAAAACCCGGCAGCGAGG	TGACGGGGGCTGTCAGGGAAT
FOXO1 (ChIP)	GGACTTGACAGATCGCAGCGAAAG	CCACGATGTCTTTGCTGAACGACG
GAPDH	GACCTGCCGTCTAGAAAAACCTGC	TCGCTGTTGAAGTCAGAGGAGACC
EMSA #1	TGGTTCTCTCTAACTGCGCTCCTGAGCCCTTCGCTGCCGG	
EMSA #2	TCGCTGCCGGCCCTGGACTTAGTGGACGGAGCGGGAGATT	
EMSA #3	GCGGGAGATTCCCGCCCAGGGGAAGAGGTTCCCACGGAGG	
EMSA #4	CCCACGGAGGGCATGCGGTCGCGGGTGCCTTTGGATTCGT	
EMSA #5	TTGGATTCGTGTGTTCAGGGAGGCCAGATAATCTCCTCCC	
EMSA #6	ATCTCCTCCCCGCGCCGGAGCCCCTGTTCCTCATTTCTAG	
EMSA #7	TCATTTCTAGCATTTTAAAAAAGGTCAAGAATACAGTGAA	
EMSA #8	ATACAGTGAATCCGGCTGGGGGCTGCCGGCTGGGTGACGC	
EMSA #9	TGGGTGACGCGCCTCTGGCTAGACCGAATGGCACAGGCTG	
EMSA #6.1	ATCTCCTCCCCGCGC	
EMSA #6.2	CTCCCCGCGCCGGAG	
EMSA #6.3	CGCGCCGGAGCCCCT	
EMSA #6.4	CGGAGCCCCTGTTCC	
EMSA #6.5	CCCCTGTTCCTCATT	
EMSA #6.6	GTTCCTCATTTCTAG	
